# Methodology for Isolation of miRNA From the Serum of Women Investigated for Pre-eclampsia

**DOI:** 10.7759/cureus.46181

**Published:** 2023-09-29

**Authors:** Flora Chamberlain, Dimitris Grammatopoulos

**Affiliations:** 1 Medicine, Warwick Medical School, Coventry, GBR; 2 Molecular Medicine, University Hospital Coventry, Coventry, GBR

**Keywords:** clinical biochemistry laboratory, next generation sequencing (ngs), developing methodology, diagnostic marker, obstetric medicine, serum mirna, circulating exosomes, pre-eclampsia

## Abstract

Background

Pre-eclampsia remains a leading cause of maternal and foetal mortality with a poorly understood pathophysiology. It can lead to a range of clinical presentations, but proteinuria and hypertension are key components of the diagnosis. These signs arise due to disordered placental implantation due to poor trophoblastic invasion, resulting in placental oxidative stress due to hypoxia. Oxidative stress triggers the release of syncytiotrophoblast microvesicles (STMBs), of which placenta-derived exosomes may be a key component. The high specificity of exosomes for their cell of origin makes them ideal candidates as diagnostic biomarkers. We are particularly interested in the miRNAs (microRNAs) contained within these exosomes, as they may give us an insight into the genomic regulation within the pre-eclamptic placenta that leads to the disease state. The development of workflows for miRNA quantitation may enable us to identify novel biomarkers.

Methods

We extracted exosomes and purified total RNA from 23 serum samples using the Norgen Plasma/Serum Exosome Purification and RNA Isolation Midi Kit. We then used the bioanalyser to determine the concentration and quality of the RNA obtained. It uses rapid electrophoresis, requires minimal sample sizes, and can assess the quality of genetic material as small as 25 bases.

Results

We have successfully isolated RNA from these samples; however, the concentration of the total RNA was too low for downstream molecular analysis. We did gain insight into how to optimise and develop the workflow so that, with each attempt, the yield increased. Our greatest concentrations were obtained by combining serum samples from multiple patients, demonstrating that we needed a higher volume to optimise the yield. Future studies should aim to obtain samples specifically for use in this research so that we can process a larger volume of serum.

Conclusions

We have also noted that there is a positive correlation between the overall concentration of total RNA and a high sFlt-1/PlGF ratio. Preliminary analysis from Illumina identified with a high degree of confidence the presence of three miRNAs, namely, mir-498(46), mir-122(1), and mir-134(41). Further work is necessary to validate these findings and should focus on the possible future role of these miRNAs as biomarkers for the early diagnosis of pre-eclampsia.

## Introduction

Pre-eclampsia is a common disorder of pregnancy with a poorly understood pathophysiology [1]. It is defined by the International Society for the Study of Hypertension in Pregnancy (ISSHP) as 'gestational hypertension accompanied by one or more of the following new-onset conditions at or after 20 weeks’ gestation: (1) proteinuria; (ii) other maternal organ dysfunction (e.g., acute kidney injury [AKI], liver involvement, neurological complications, or haematological complications); (3) uteroplacental dysfunction (such as foetal growth restriction, abnormal umbilical artery Doppler waveform analysis, or stillbirth) [1].

The multi-system nature of pre-eclampsia makes its aetiology difficult to identify. It is understood that the underlying pathogenesis is based on disordered placentation due to poor trophoblastic invasion, resulting in insufficient remodelling of the maternal spiral arteries [2,3]. This causes poor placental perfusion, and the resulting oxidative stress triggers the release of syncytiotrophoblast microvesicles (STBMs) from the syncytial layer of the placenta [4]. The release of STBMs may lead to an altered maternal systemic inflammatory response because they contain pro-inflammatory cytokines as well as exosomes, foetal DNA, and other factors [4]. Other contributing factors to the pathophysiology of pre-eclampsia include insufficiency of gestational immune tolerance, leading to an immune response to foetal material, and a slowed migration of cytotrophoblasts when they invade the uterine spiral arteries [5].

It is vital that we continue to gain an improved understanding of the molecular basis of pre-eclampsia, as it remains a leading cause of maternal mortality, causing 70,000 maternal deaths and 500,000 foetal deaths worldwide every year [6]. The management of pre-eclampsia includes anti-hypertensive medications and magnesium sulphate for the prevention of seizures, but ultimately the only cure for pre-eclampsia is the delivery of the baby [4]. There has been research into preventative therapies for pre-eclampsia, including weight management, stress reduction, aspirin, calcium supplementation, and various antioxidant vitamins, with promising early data for low-dose aspirin and calcium supplementation [7-9]. These may be possible avenues for early management and prevention of disease progression if we were able to predict cases of pre-eclampsia before they developed.

Since pre-eclampsia is most common during the third trimester, routine antenatal care for this period incorporates screening for the disorder through blood pressure monitoring and urinalysis to screen for hypertension and proteinuria [4]. In recent years, further investigations for high-risk pregnancies have been introduced, including Doppler ultrasound of the uteroplacental circulation and testing for circulating biomarkers [4]. Specifically, the ratio of soluble fms-like tyrosine kinase-1 (sFlt-1) to placental growth factor (PIGF) is one blood test currently used, in combination with clinical signs and symptoms, to diagnose suspected pre-eclampsia [10]. The sFlt-1/PlGF ratio can only be used to rule pre-eclampsia in or out in the short term, so a negative test can only be used to exclude pre-eclampsia for one week [10]. Therefore, the blood test is only recommended by NICE for use in suspected pre-eclampsia and is not currently used in early antenatal care to predict possible future cases.

The sFlt-1/PlGF ratio has been evaluated as a test to predict the presence of pre-eclampsia, but Zeisler et al. found that a ratio above 38 had a positive predictive value of 36.7% and a sensitivity of 66.2% for a diagnosis of pre-eclampsia within four weeks [11]. The test is, therefore, not recommended by NICE for use in routine antenatal testing to predict pre-eclampsia. The main function of the test is to rule out the disorder instead. It is our hope that if we had a definitive diagnostic test for pre-eclampsia early in pregnancy, we could delay the onset of the disorder and minimise fatalities.

Evidence that placenta-derived exosomes may be a key component of STBMs has identified them as a possible biomarker for pre-eclampsia. Exosomes are vesicles released from cells, containing cell-specific genetic material, proteins, and lipids. Their high specificity for their cell of origin makes them ideal candidates as diagnostic biomarkers [12]. Particularly of interest is the miRNA (microRNA) fingerprint of the exosomes. MiRNAs are short, non-coding strands of single-stranded RNA, approximately 20 nucleotides in length. Although they do not code for proteins, they are increasingly recognised as having vital roles in the regulation of gene expression as they can interact with messenger RNAs to alter their translation into proteins [13]. The many regulatory roles of miRNAs in cancer and other pathologies are only just beginning to be uncovered, and there is much to explore regarding their possible applications in diagnostics and as therapeutic targets [13]. Since each tissue type has its own unique proteome to help it deliver its specific function, it follows that each tissue has a unique RNA fingerprint and a unique set of miRNAs to regulate its expression. We know that exosomes are released with STBMs in response to oxidative stress in pre-eclampsia [3], and we hope that by investigating the miRNA fingerprint of the exosomes released by the pre-eclamptic placenta, we can identify miRNAs with important roles in the pathophysiology of pre-eclampsia and possibly miRNAs that are expressed with enough consistency to be used as a biomarker for the prediction of pre-eclampsia.

Previous studies have identified specific miRNAs that may be of interest when investigating biomarkers of pre-eclampsia by carrying out microarrays to look at the differential expression of miRNAs between normal and pre-eclamptic pregnancies [14]. A number of miRNAs may be of interest: miR-155 [15], miR-210 [16], and a miRNA cluster located on chromosome 19, which incorporates miR-517-5p, miR-520a-5p, and miR-525-5p [17]. All of these candidates have previously been identified as being differentially expressed in pre-eclampsia. We hope to recreate this finding and clarify the consistency with which these miRNAs are raised. If all or some of these miRNAs are consistently altered in the pre-eclamptic state, we would want to validate them as biomarkers of pre-eclampsia, with the view to seeing them incorporated into routine antenatal care for the screening of pre-eclampsia.

The aim of this study is, therefore, to develop a workflow through which exosomes and miRNA can be extracted from serum or plasma. This workflow will consist of the isolation of placental exosomes from the blood of pregnant people with and without pre-eclampsia. We will then extract the exosomal RNA, hopefully including the miRNAs of interest. We will verify the presence of RNA with the bioanalyser, which determines the quality and quantity of the RNA in our samples. Iterative refinements of the protocol will hopefully improve the concentration of these samples so that they are suitable for downstream molecular applications. We then hope to compare the exosomal miRNA profiles of the different samples through next-generation sequencing (NGS) to identify consistent biomarkers of pre-eclampsia.

The main method of molecular analysis that we would like to use is NGS. This involves the development of RNA libraries through the generation of cDNA from our extracted RNA [18]. This would tell us the specific sequence of the miRNAs isolated so that we can learn about their role on a molecular level and hopefully about the pathophysiology of pre-eclampsia. The data generated will be subject to bioinformatic analysis to identify new miRNAs of interest that may be investigated in the future as biomarker candidates. The NGS analysis will also serve as proof of principle, either demonstrating that we have successfully isolated the RNA or showing that we will need to adjust our protocol to increase the yield.

The concentrations of the RNA alone may also be able to give us information about a possible pre-eclampsia diagnosis. The use of RNA biomarkers is increasingly being incorporated into the diagnosis of certain cancers, just by looking at the levels of non-coding RNAs [13,19]. Given our understanding of the pathophysiology of pre-eclampsia, an increased level of the miRNA biomarkers could indicate pre-eclampsia. Increased levels of placental hypoxia may lead to a greater release of STBMs into the circulation, more exosomes, and a greater concentration of exosomal miRNAs. Therefore, the concentration of miRNA alone could be sufficient for diagnosis. The development of a simple screening test that could identify patients at risk of pre-eclampsia so that they can be supported and observed during early pregnancy could massively improve both foetal and maternal outcomes.

This work was previously presented as a poster presentation at the 2023 INSPIRE National Graduate Entry Medicine Research Conference in Coventry, UK, on September 2, 2023.

## Materials and methods

Patients and sample collection

This study was approved by the clinical research ethics committee of the Arden Tissue Bank (approval date: June 13, 2022; ethics number: 18/SC/0180). We used 23 anonymised samples, which were surplus to requirements from routine samples taken for the purpose of investigating the patients for pre-eclampsia or undergoing routine first-trimester antenatal testing. All tissue samples were provided by the Arden Tissue Bank. They were secured and stored on our behalf by the pathology department at University Hospital Coventry and Warwickshire.

Exosome isolation and RNA purification

Exosomal miRNAs were purified from the serum of patients under investigation for pre-eclampsia or undergoing routine first-trimester antenatal testing. The exosomes were isolated, and the exosomal RNA was extracted using a Norgen Plasma/Serum Exosome Purification and RNA Isolation Midi Kit [20] according to the manufacturer’s instructions. The RNA isolated includes the miRNAs of interest, as well as other exosomal RNA and some cell-free RNAs that are free in the serum. The Norgen Biotek kit was chosen over other options because there is no requirement for specialist instruments or training, and some comparison studies show that it yields a higher concentration of miRNAs than comparable kits [21]. The protocol of this kit involves the use of a silicon carbide resin column to purify and concentrate exosomes, followed by their lysis and the purification of protein-free RNA.

Bioanalyser

The Agilent 2100 Bioanalyser was used to perform an RNA pico assay with the Agilent RNA 6000 Pico Kit. This involves the separation of nucleic acid fragments by size using electrophoresis, the subsequent generation of a size profile, and an assessment of the integrity and concentration of the RNA. The quality and concentration of the RNA were determined according to the manufacturer’s protocols [22].

Next-generation sequencing

Sequencing libraries were generated using the Illumina TruSeq® Small RNA Library Prep Kit, according to the manufacturer’s instructions [23]. The iSeq Sequencing System was then used to carry out the NGS.

## Results

Sample concentrations

Table 1 shows the concentrations of total RNA obtained from each sample and the corresponding sFlt-1/PlGF ratio for the serum samples incorporated into each extraction. In total, we completed 23 RNA extractions from various samples.

**Table 1:** Bioanalyser results RNA concentrations for the 23 extractions completed. For comparison, the sFlt-1/PlGF ratio for the samples in each extraction are included. The sFlt-1/PlGF ratios which are above 33 are over the rule out cut-off for pre-eclampsia [10].

Sample	Concentration (pg/μl)	sFlt1/PlGF ratio
1	185	15
2	326	22
3	230	16
4	25	30
5	21	12
6	40	13
7	279	-
8	716	-
9	416	-
10	318	-
11	456	-
12	341	-
13	335	-
14	378	14
		22
		18
15	333	23
		29
		10
16	240	22
		45
		37
17	203	10
		8
		19
18	229	17
		22
19	187	18
		9
20	1026	34
		30
		27
21	668	13
		22
		27
22	1131	35
		30
		44
23	663	88

Samples 1-6 were from the initial attempt, using only one patient’s serum per extraction, giving very low concentrations of RNA that were insufficient for downstream applications.

For the next two, we decided to compare routine first-trimester samples with samples from patients specifically being investigated for pre-eclampsia. The RNA concentration of the serum under specific investigation is much higher. If our hypothesis is correct that increased hypoxia in pre-eclampsia would lead to increased STMBs and an increased RNA concentration, this could be reflected in this data. Unfortunately, we do not have the sFlt-1/PlGF ratios for these patients, so we cannot draw this conclusion.

Then we compared fresher samples with those that had been frozen for a longer time (samples 9-13). Samples 9-10 were the fresh samples, and samples 11-13 were the frozen ones. There is no discernible pattern in the RNA concentrations between the fresh and frozen samples, a conclusion that is supported by the literature [24].

Samples 14-22 were more combined samples of patients under investigation for pre-eclampsia but without a confirmed diagnosis. Sample 23 contained serum from only one patient who had a confirmed diagnosis of pre-eclampsia. Of particular interest are the samples that contain serum with an sFlt-1/PlGF ratio greater than 33, which is the cut-off for ruling out pre-eclampsia between weeks 20 and birth [10]. Samples 20-23 were all processed together as a batch under identical circumstances. Samples 20 and 22 have much greater concentrations compared to sample 21, the only sample that did not include serum with an sFlt-1/PlGF ratio greater than 33. This is supportive of our hypothesis that an increased RNA concentration will be seen in patients with pre-eclampsia but will need to be reproduced in later experiments.

Also of note is sample 23, the only sample in this group that contained serum from only one patient with a confirmed pre-eclampsia diagnosis. This sample’s RNA concentration is comparable to that of sample 21, which contains the serum of three patients in whom pre-eclampsia can be ruled out. This further demonstrates that an increased overall RNA concentration may be caused by the release of STMBs in pre-eclampsia. Although further validation of this result will be required, we hope it may reflect a possible future role for miRNAs in the diagnosis of pre-eclampsia, as this correlation is quite striking.

Bioanalyser results

**Figure 1:**
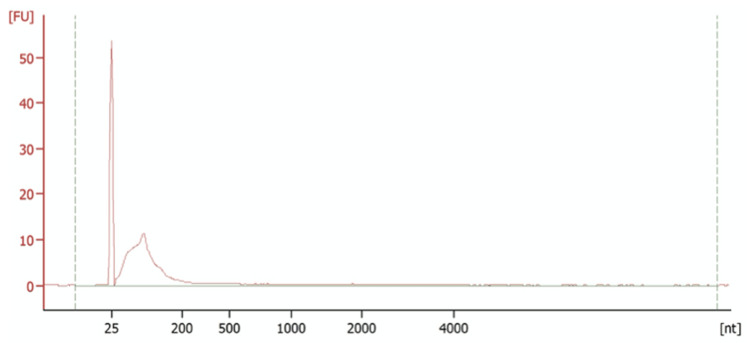
Bioanalyser trace for sample 2 The reference marker is at 25 nt and our sample is illustrated by the broad peak ranging from 25-200 nt. The concentration of this sample was 236 pg/μl.

Figure 1 shows the bioanalyser output for sample 2, an example of the first set of samples that were sent for analysis in the bioanalyser. The peak at 25 nt corresponds to the reference marker. The broad peak between 25 and 200 nt indicates the RNA that we have isolated, demonstrating that we have successfully isolated RNAs in this size range. This will include some miRNAs, which average around 22 nucleotides in length. Downstream analysis would confirm the identity of these miRNAs; however, the concentration of sample 2 was only 326 pg/μl, which is insufficient to run NGS as the Illumina assay requires a minimum concentration of 10-50 ng/μl. We therefore needed to try various methods to increase the concentration of the samples.

**Figure 2:**
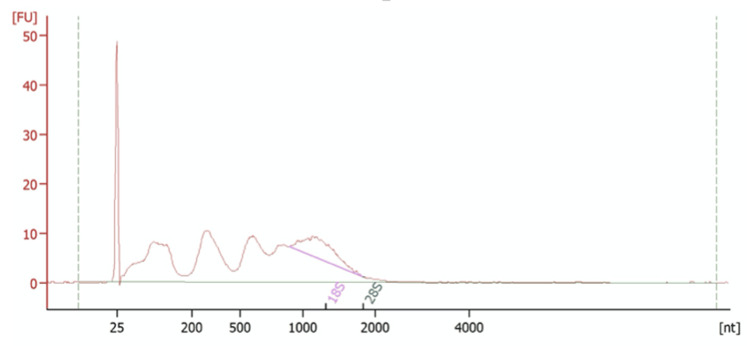
Bioanalyser trace for sample 20 The reference marker is at 25 nt and our sample is illustrated by the broad peak ranging from 25-2000 nt. The concentration of this sample was 1026 pg/μl.

Figure 2 shows the bioanalyser output for sample 20, a combined sample containing serum from three separate patients. The concentration of this sample was 1026 pg/μl, one of the highest concentrations obtained. The peak at 25 nt corresponds to the reference marker. There are multiple peaks between 25 and 2000 nt, corresponding to our sample. This range includes the miRNA range but also lncRNA (long non-coding RNA), which may also be of interest in the regulation of pre-eclampsia. Unfortunately, the peak in the 25-200 range has a height comparable to that of sample 2, indicating that the concentration of miRNA is unlikely to be much higher in this sample. However, the overall concentration of this sample being higher makes it more suitable for downstream applications, so we are more likely to be able to examine the miRNA in more detail.

**Figure 3:**
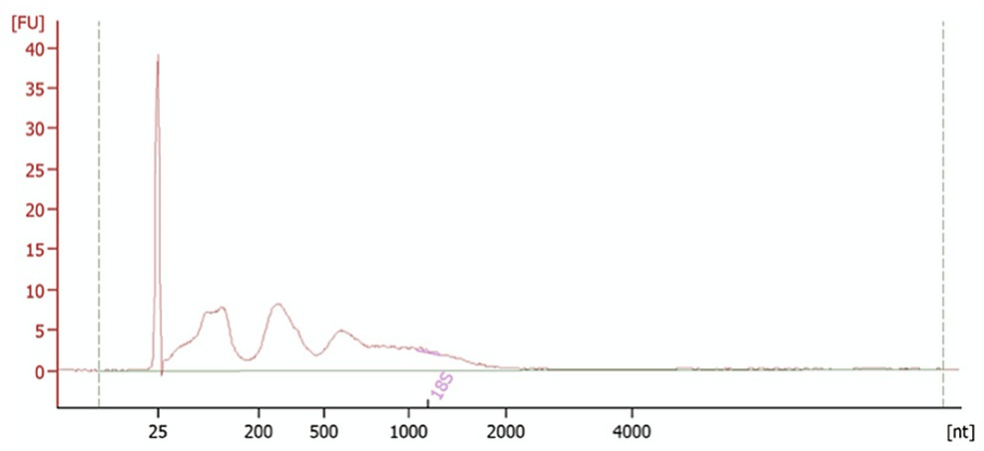
Bioanalyser trace for sample 23 The reference marker is at 25 nt and our sample is illustrated by the broad peak ranging from 25-2000 nt. The concentration of this sample was 663 pg/μl.

Figure 3 shows the bioanalyser output for sample 23, which has been of particular interest as the only sample with a confirmed pre-eclampsia diagnosis. The concentration of this sample was 663 pg/μl, which was comparable to a sample containing the serum of three patients in whom pre-eclampsia can be ruled out. The peak at 25 nt corresponds to the reference marker. There are multiple peaks between 25 and 2000 nt, corresponding to our sample. This range also includes both the miRNA range and the lncRNA range. Particularly, there are two discrete peaks around 75-150 nt and 200-450 nt, which appear more distinct with less background noise than the other samples. In future work, it will be interesting to investigate the specific identity of the RNAs at these peaks.

Next-generation sequencing results

NGS was run on sample 22, the most concentrated sample at 1131 pg/μl. Unfortunately, the bioinformatic analysis will not return in time for the sequence data to be included in this report, but we do have preliminary data available.

The total yield of the sequenced output was 0.1 GB (gigabases). A Q (quality) score is assigned to each base to indicate the quality of the reading. If a score of Q30 is assigned to the base, there is a 1 in 1000 probability of that base being called incorrectly [25]. Sample 22 returned a Q30% of 71.7%, so 71.7% of the bases have a Q score of 30 or higher. A Q30% score of 70-80% or higher is considered acceptable.

The percent passing filter (%PF) indicates the percentage of reads that pass Illumina’s internal quality filtering procedure, called the 'chastity filter', which removes the least reliable clusters from the results. The %PF generated for sample 22 is 17.3%, so a very low percentage of reads have passed the chastity filter.

Inadequate concentrations of the RNA library may have led to this discrepancy between the Q30% and %PF. The %PF may be low because of the low concentration of the RNAs, making the clusters less discernible, while the high Q30% reflects that those clusters that did pass the chastity filter are usable. Hopefully, in future research, protocol modifications will increase the yield of RNA so that the NGS reads are of superior quality. Nevertheless, preliminary analysis from Illumina identified with a high degree of confidence the presence of 3 miRNAs, namely, mir-498(46), mir-122(1), and mir-134(41).

## Discussion

In our efforts to increase the yield of the RNA extraction, we tried using larger sample volumes by combining multiple samples for one extraction. We also tried processing fresher samples in case the stability of the RNA was being affected by long-term freezing or repeated thawing. However, this is harder to organise since the samples are not taken specifically for our study. A closer examination of the literature demonstrates that multiple freeze-thaw cycles do not negatively impact the stability of miRNAs [24]. Ultimately, our data for samples 9-13 demonstrate little difference between fresher samples and those which have been frozen for a longer period.

Another option that was considered was to use the miRNeasy Serum/Plasma Advanced Kit from Qiagen [26] to isolate the smaller RNAs from the total RNA samples that we have obtained. This would remove some remaining proteins and use multiple washes to remove other contaminants. We hope that this will give a cleaner signature on the bioanalyser trace and facilitate the sequencing of our miRNAs of interest by simplifying the input. Given the nature of this project, there was insufficient time to order and carry out this kit, but in future research into miRNAs, we would recommend this to purify the miRNAs and reduce background noise. However, one limitation may be that this would reduce the concentration of the samples, so the downstream applications are limited.

Combining multiple samples to increase the overall concentration was a successful approach. We did see an overall increase in concentrations with the combined samples and higher volumes. The limitations of this approach will come in the downstream analysis. Since we did not know the sFlt-1/PlGF ratios of the samples before they were combined, some contain miRNA from patients both with and without pre-eclampsia. Therefore, when we sequence the samples, it will be hard to distinguish which miRNA is specific to the pathology. In future work, we would want to obtain samples specifically for use in this research so that we could process a larger volume of serum, since using leftovers from the lab often meant that we received less than a millilitre to process. Unfortunately, the time limitation and ethical implications meant that this was beyond the scope of this project.

Alternatively, we could use the Norgen mini kit instead of the midi kit that we used. This would mean fewer reagents being added to the serum, hopefully making it less dilute and increasing the yield at each stage of the purification.

## Conclusions

We have successfully tested various steps, identified limitations, and generated information useful for the development of a successful workflow for the purification of exosomal RNA from serum. We have successfully demonstrated the presence of miRNA in these samples through the bioanalyser traces. We were limited in our ability to carry out downstream molecular analysis due to the low concentrations obtained.

Our findings indicate the need for higher volumes of serum in this workflow to optimise RNA concentration and the continued refinement of this protocol. We have also identified a positive correlation between total RNA concentration and raised sFlt-1/PlGF ratios. A future investigation of this finding may illustrate the role of regulatory RNAs in pre-eclampsia pathogenesis.
